# Safest Time to Resume Oral Anticoagulation in Patients with Traumatic Brain Injury

**DOI:** 10.7759/cureus.2920

**Published:** 2018-07-03

**Authors:** Yana Puckett, Kelly Zhang, Jay Blasingame, Jessica Lorenzana, Shamini Parameswaran, Steven E Brooks, MD, FACS, Benedicto C Baronia, John Griswold

**Affiliations:** 1 Surgery, Texas Tech University Health Sciences Center, Atlanta, USA; 2 Surgery, Texas Tech Health Sciences Center, Lubbock, USA; 3 Surgery, Methodist Health System, Dallas, USA; 4 Medicine, Northern California Kaiser Permanente, San Francisco, USA; 5 Thoracic Surgery, Yale-New Haven Hospital, New Haven, USA; 6 Trauma and Acute Care Surgery, Texas Tech University Health Sciences Center, Lubbock, USA; 7 Neurosurgery, Texas Tech University Health Sciences Center, Lubbock, USA; 8 General Surgery, Texas Tech Health Sciences Center, Lubbock, USA

**Keywords:** anti-platelet therapy, anti-coagulation therapy, trauma, traumatic brain injury, thromboembolism, death, stroke

## Abstract

Objective: There is no standard protocol to guide the optimal time to resume anti-clotting agents after traumatic brain injury (TBI) in patients with a continued indication for anticoagulation/antiplatelet therapy (AAT). This study develops baseline data supporting a future prospective cohort study. We predict that there will be significantly decreased adverse events when AAT is started on or after Day 7.

Methods: A retrospective chart review of 256 patients was performed. Patients admitted to a level I trauma center in West Texas between January 1, 2009, and December 31, 2012, on anti-clotting agents (specifically acetylsalicylic acid, coumadin, and/or clopidogrel) and who suffered a TBI were included. Patient metrics included admission coagulation studies, type of TBI and treatment, and time to continuation of AAT. Outcomes were assessed using follow-up appointment data. The primary outcome was death (mortality). Secondary outcomes included myocardial infarction, stroke, re-bleed, venous thromboembolism, and pneumonia.

Results: A total of 256 patients met the inclusion criteria. However, only 85 patients on AAT presented for the six-month follow-up. Time to AAT resumption varied from immediate to 31 days. Out of the 85 patients, 32 patients never resumed AAT, 32 patients were restarted on AAT medication in less than seven days, 10 patients restarted medication between seven and 14 days, and 11 patients restarted AAT in more than 14 days. Adverse events occurred most infrequently in the AAT group resuming therapy between seven and 14 days (10%). Adverse events were most prevalent in the AAT group that never resumed therapy (68.8%).

Conclusion: While most studies suggest that the safest time for resuming AAT lies between three and 10 days, our study revealed that adverse events were minimized in patients on AAT between seven and 9.5 days.

## Introduction

Over the past decade, the number of patients on oral anticoagulation and/or oral antiplatelet therapy (AAT) has continued to rise [1-5]. Additionally, new drugs with unclear mechanisms of reversal are increasingly available [6-9]. This has prompted increased demand for physician knowledge regarding the management of these anti-clotting agents, especially in traumatic brain injury (TBI). This event accounts for an estimated 275,000 hospitalizations each year in the United States [10]. The rate of TBI-related emergency department (ED) visits has risen sharply from a rate of 457.5 per 100,000 visits in 2007 to a rate of 715.7 per 100,000 visits in 2010. Patients on anti-clotting agents represent an increasing portion of this population [10].

The management of patients on long-term anticoagulation/antiplatelet therapy (AAT) may be challenging due to an increased risk of serious injury from resuming medications too early or too late and worse outcomes are associated in patients with TBI and midline shift [11-18]. The anticoagulant or antiplatelet medications are often indicated for pre-existing medical conditions or subsequent to cardiovascular stent placement. Considering the risks and benefits of discontinuing the medications and following the patient for complications related to hypercoagulability are crucial aspects of these patients’ care.

Narrowing the timeframe for restarting AAT to a more precise window should be a priority. Consistency in this decision-making is sorely lacking and is reflected in the literature. The resumption of AAT has been guided by the consensus of treating teams, including the surgeon, critical care physician, and cardiologist.

Despite the increasing numbers of both patients and new anti-clotting agents, there is still no established or affirmed protocol to suggest when patients on long-term AAT with diagnosed TBI should resume their AAT. This study aims to develop a protocol that will endorse a more uniform approach to AAT resumption, as multiple studies show that patient outcomes improve when protocols exist to guide physicians with evidence-based best practice [19-22].

## Materials and methods

This retrospective review was approved by the Texas Tech University Health Sciences Center Institutional Review Board For The Protection of Human Subjects, IRB #: L14-111; Submission Reference #: 052438. Subjects were identified using the discharge database at University Medical Center, a tertiary referral hospital and Level I trauma center. Inclusion criteria consisted of patients aged 18-89 years with head trauma who were taking coumadin, acetylsalicylic acid, clopidogrel, or a combination of these medications and were diagnosed with a traumatic brain injury (defined as concussion, subdural hematoma, epidural hematoma, subarachnoid hemorrhage, and intracranial hemorrhage) and admitted between January 1, 2009, and May 18, 2014.

Patients admitted to our hospital with head trauma were examined and/or admitted by the trauma service. Imaging and lab workup were directed by the attending physician. Neurosurgery service was consulted and recommendations followed for all patients with identified TBI. These patients were started on anti-epileptic medication and were either treated nonoperatively or received craniotomy. Anticoagulation medications were discontinued upon admission. Various AAT reversal agents, including fresh frozen plasma, prothrombin complex concentrates, and platelets, were used to correct the coagulopathy of patients at the time of treatment of these patients. Treatment time varied from patient to patient, and it is possible some patients received AAT reversal agents upon admission.

Medical records were reviewed for the following: age, sex, race, mechanism of head trauma, anticoagulant medication, length of stay, type of intracranial bleeding disorder, Glasgow Coma Scale (GCS); use of illicit drugs, international normalization ratio (INR), cranial surgical interventions, length of time until AAT was resumed, and adverse outcomes associated with discontinuing AAT. Outcomes were assessed using six-month follow-up clinic visit records and the following conditions were identified: hemorrhagic or thromboembolic events, i.e., stroke, myocardial infarction (MI), venous thromboembolism (VTE), pulmonary embolism (PE), or re-bleed. Medians were used for some continuous data due to this data being not normally distributed. Since the data are not normally distributed, a Kruskal-Wallis one-way analysis of variance (ANOVA) was used to determine the statistical significance of this study.

## Results

Out of 256 patients who sustained a traumatic brain injury (TBI) and were on anticoagulation/antiplatelet therapy (AAT) on arrival to our Level I trauma center, 85 patients met inclusion criteria. Of these, 44.7% were female and the median age of the patients was 75 years. The median length of stay for all patients was three days. Caucasian patients made up 71.8% of the studied population, Hispanics 23%, and African-Americans 4.7%. Those classified as "Other" comprised 1.5% of the population. On arrival at the hospital, 64% of the patients were on coumadin, 28.2% were on aspirin, and 25.9% were on clopidogrel. Of these patients, 10.6% were on both aspirin and clopidogrel and 4.7% were on both acetylsalicylic acid and coumadin (Table 1).

**Table 1:** Demographics and baseline characteristics of patients OAC (oral anticoagulation), IQR (interquartile range), AE (adverse events), ASA (aspirin)

	Overall n=85	No OAC n =32	< 7 days n=32	7-14 days n=10	>14 days n=11
Age, median(IQR)	75 (60.5 – 82.0)	80 (69.5 – 86.0)	66.5 (50.2 – 77)	74 (62.5 – 78.8)	78 (70 – 87)
Female, n (%)	38 (44.7)	16 (50.0)	11 (34.4)	6 (60.0)	5 (45.5)
LOS, median (IQR)	3 (1 – 5.5)	4 (2 – 4)	2 (1 – 4)	2 (1 – 7.5)	5 (3 – 9.0)
Race, n (%)					
White	61 (71.8)	24 (75.0)	21 (65.6)	8 (80.0)	8 (71.8)
Hispanic	20 (23.5)	5 (15.6)	11 (34.4)	2 (20.0)	2 (23.5)
Black	4 (4.7)	3 (9.4)	0 (0.0)	0 (0.0)	1 (4.7)
Coumadin, n (%)	55 (64.7)	20 (62.5)	23 (71.9)	8 (80.0)	4 (36.4)
ASA, n (%)	24 (28.2)	12 (37.5)	4 (12.5)	4 (40.0)	4 (36.4)
Plavix, n (%)	22 (25.9)	8 (25.0)	7 (21.9)	1 (10.0)	6 (54.5)
Plavix & ASA, n (%)	9 (10.6)	5 (15.6)	1 (3.1)	0 (0.0)	3 (27.3)
Coumadin & ASA, n (%)	4 (4.7)	2 (6.3)	0 (0.0)	2 (20.0)	0 (0.0)
Midline shift, n (%)	23 (27.1)	16 (50.0)	3 (9.4)	0 (0.0)	4 (36.4)
GCS on Day 1, median (IQR)	15 (13 – 15)	14 (3.25 – 15)	15 (15 – 15)	15 (15 – 15)	14 (14 – 15)
INR on Day 1, median (IQR)	1.76 (1.07 – 2.82)	1.79 (1.07 – 2.88)	1.74 (1.07 – 2.83)	2.05 (1.50 – 3.91)	1.08 (1.03 – 5.59)
Anticonvulsant, n (%)	32 (37.6)	13 (40.6)	9 (28.1)	6 (60.0)	4 (37.6)
LOC, n (%)	33 (38.8)	16 (50.0)	10 (31.3)	4 (40.0)	3 (27.3)
Trauma, n (%)	15 (17.6)	5 (15.6)	4 (12.5)	2 (20.0)	4 (36.4)
Fall, n (%)	58 (68.2)	25 (78.1)	21 (65.6)	7 (70.0)	5 (45.5)
MVC, (%)	15 (17.6)	5 (15.6)	7 (21.9)	1 (10.0)	11 (12.9)

A non-contrast head computed tomography (CT) scan showing a midline shift was found in 27.1% of the studied population. The average overall GCS was 13. The median international normalized ratio (INR) on Day 1 was 1.76. Ground-level fall (GLF) was the most common cause of brain injury, occurring in 68.2% of the patients (Table 1).

Out of 85 patients, 32 were discontinued on AAT indefinitely. The average age of this group was 80 years, with 50% men and 50% women. Of these patients, 62% were taking coumadin, 37.5% were taking acetylsalicylic acid, and 25% were taking clopidogrel bisulfate. Of these, 15.6% were on both clopidogrel and aspirin combined and 6.3% were on coumadin and aspirin combined. Ground-level fall was the most common trauma mechanism (78.1%). Fifty percent had a “midline shift (Tables 1-2).

**Table 2:** Outcomes by type of bleed (n = 85) SDH (subdural hemorrhage), SAH (subarachnoid hemorrhage), OAC (oral anticoagulation), MI (myocardial infarction), AE (adverse events), PE (pulmonary embolism), DVT (deep vein thrombosis)

	SDH n = 33	SAH n = 22	Closed Head Injury n = 26	Intraparenchymal Bleed n = 19	Epidural Hematoma n = 1
Days to receiving OAC, median(IQR*)	1 (0 – 5.5)	2 (0 – 7.5)	1 (0 – 2.5)	1 (0 – 11)	0
Complications**, n (%)					
Death	16 (48.5)	4 (18.2)	6 (23.1)	9 (47.4)	1 (100.0)
Stroke	0 (0.0)	1 (4.5)	0 (0.0)	0 (0.0)	0 (0.0)
MI	5 (15.2)	1 (4.5)	1 (3.8)	3 (15.8)	0 (0.0)
Re-bleed	4 (12.1)	1 (4.5)	2 (7.7)	1 (5.3)	1 (100.0)
Pneumonia	2 (6.1)	0 (0.0)	1 (3.8)	0 (0.0)	0 (0.0)
Number of AEs					
0	17 (51.5)	17 (77.3)	19 (73.1)	10 (52.6)	0 (0.0)
1	7 (21.2)	3 (13.6)	4 (15.4)	5 (26.3)	0 (0.0)
2	8 (24.2)	2 (9.1)	3 (11.5)	4 (21.1)	1 (100.0)
3	0 (0.0)	0 (0.0)	0 (0.0)	0 (0.0)	0 (0.0)
4	1 (3.0)	0 (0.0)	0 (0.0)	0 (0.0)	0 (0.0)

Overall, 22 people (68.8%) in the group that did not resume anticoagulation/antiplatelet therapy had complications. Stroke occurred in one person (3.1%), myocardial infarction occurred in five people (15.6%), re-bleed occurred in seven people (21.9%), and pneumonia occurred in two people (6.3%). The median length of stay for this group was four days (Table 2).

A total of 32 patients were started on AAT within seven days, with Day 1 as the median day for AAT resumption. In this group, no stroke, re-bleed, or pneumonia was found. However, a myocardial infarction occurred in one person (3.1%). The average age in this group was 66.5 years with 34.4% being female. The median length of stay for this group was two days. Only 9.4% had a midline shift. Again, GLF was the most common injury mechanism (65.6%) (Table 3).

**Table 3:** Outcomes by days to OAC use OAC (oral anticoagulation), AE (adverse events), PE (pulmonary embolism), DVT (deep vein thrombosis)

	Overall n=85	No OAC n=32	< 7 days n=32	7-14 days n=10	>14 days n=11
Days to receiving OAC, median (IQR*)	1 (0 – 5.5)	-	1 (1 - 2)	7 (7 – 9.5)	30 (24 – 31)
Complications**, n (%)					
Death	27 (31.8)	22 (68.8)	2 (6.3)	1 (10.0)	2 (18.2)
Stroke	1 (1.2)	1 (3.1)	0 (0.0)	0 (0.0)	0 (0.0)
MI	7 (8.2)	5 (15.6)	1 (3.1)	0 (0.0)	1 (9.1)
Re-bleed	8 (9.4)	7 (21.9)	0 (0.0)	0 (0.0)	1 (9.1)
Pneumonia	2 (2.4)	2 (6.3)	0 (0.0)	0 (0.0)	0 (0.0)
Number of AEs					
0	56 (65.9)	8 (25.0)	30 (93.8)	9 (90.0)	9 (81.8)
1	15 (17.6)	13 (40.6)	1 (3.1)	1 (10.0)	0 (0.0)
2	13 (15.3)	10 (31.3)	1 (3.1)	0 (0.0)	2 (18.2)
3	0 (0.0)	0 (0.0)	0 (0.0)	0 (0.0)	0 (0.0)
4	1 (1.2)	1 (3.1)	0 (0.0)	0 (0.0)	0 (0.0)

A total of 10 patients resumed AAT between Days 7-14; with the median as Day 7 and an interquartile range of 7-9.5 days. This group had no occurrences of stroke, MI, re-bleed, or pneumonia. The average age of this group was 74, and 60% were female. The median hospital length of stay was two days with an interquartile range of 1-7.5. There were no midline shifts in this group. GLF was the most common injury mechanism (70%). There was one mortality in this group (Table 3).

A total of 11 patients resumed anticoagulation/antiplatelet therapy after 14 days. Median AAT resumption in this group was Day 30, with an interquartile range of days 24-31. One person suffered from an MI and another suffered a stroke. Overall, there were two complications in this group. Four patients sustained a midline shift on CT scan, and the median length of stay was five days. Ground level fall was the most common injury mechanism (45.5%) (Table 3).

## Discussion

Patients with traumatic brain injury (TBI) who are on anticoagulation/antiplatelet therapy (AAT) challenge the treating physician, as these patients are at risk of life-threatening thrombotic complications and hemorrhagic complications. Our study attempts to retrospectively evaluate the safest time for AAT resumption while minimizing morbidity and mortality.

Previously, no guidelines have been established or affirmed that might help with this decision. The literature provides disparate answers regarding the optimal time period for the resumption of AAT. One study favors the resumption of AAT between five and 10 days [23]. However, no additional direction is provided for differences in severity of injury or midline shift due to a lack of statistical power.

Aguilar et al. (2007) performed a literature review from 1996 to 2006 and examined the safest time for AAT resumption in TBI patients. The authors could not find any prospective randomized or cohort trials to validate the suggested AAT resumption times promoted in smaller trials. However, this study combined the results of small case series with expert opinion. An expert was defined as “Someone with involvement with one or more publications on the specific topic or who had personal experience treating warfarin-associated intracerebral hemorrhage (ICH).” Unfortunately, the study does not identify the safest time to resume AAT in unstable patients or those at high risk for re-bleed or thromboembolic events.

The study herein supports AAT resumption between Day 7 and Day 9. Noteworthy in this group were the absence of a midline shift and a short median hospital length of stay (two days), which may indicate that these patients had more benign head traumas. However, due to lack of statistical power, a statistical significance could not be found for AAT resumption times (Tables 1-3). But, these data are a start in creating protocol guidelines for stable TBI patients.

We found that the worst outcome was seen in those who were never restarted on AAT. This group was associated with the worst head injuries with the majority having a midline shift on CT scan and the mean age of this cohort was older as well. It may be that worse outcomes appear in this group not because these patients did not re-start ATT but because the patients themselves were critically ill and succumbed to their injuries.

There were six thrombotic complications in this cohort. Death occurred in 22 out of 32 patients in this group. Five of the 32 suffered an MI and one suffered a stroke. There were a total of seven re-bleeds in this group despite complete discontinuation of AAT. These results are conflicting and hard to interpret due to the small number of patients. It is hard to say that the cause of death and the poor outcome of these patients can be attributed to the status of AAT resumption. A 2010 study by Hawryluk et al. found that in patients with central nervous system hemorrhage, hemorrhagic complications were common if anticoagulation was resumed within 72 hours while thromboembolic complications were more common thereafter. From this study, it is suggested that AAT resumption should not be attempted before three days [24].

Of interest, the third cohort analyzed in our study was comparable in severity of TBI to the first cohort in which OAC was held indefinitely. This cohort had fewer patients (only 11) but the OAC was restarted sometime between 24 and 31 days. Compared to the cohort in whom OAC was held indefinitely, this cohort had a slightly smaller midline shift associated with a CT scan: 36.4% versus 50% but with their median age being similar. The number of adverse events in this cohort was two (18.2%), with one patient suffering an MI and another suffering a brain re-bleed.

There are several limitations to this study. The patients were reviewed retrospectively and were managed without any specific protocol. Most of the decisions as to when to restart OAC were made by different physicians based on the clinical picture and the opinions of other consulting physicians, such as cardiologists, critical care physicians, and neurosurgeons. As such, treatments for a given grade or type of TBI were not consistent. Our study did not account for other injuries in patients with TBI. The results of this study lack statistical power secondary to a lack of a sufficient number of patients. The paucity of patients in the study was due in part to a lack of follow-up, as patients with no follow-up were excluded from the study. In addition, the nationwide switch from paper charting to electronic health records resulted in limited information and some lost information (i.e., records not scanned) from before 2011. This study, being a retrospective review, is a limitation in and of itself.

Future studies need to be performed to assess this important question. We recommend a prospective study with a six-month follow-up period for the assessment of outcomes. In this future prospective study, we will also break down traumatic brain injury (TBI) into groups based on severity at presentation to the hospital. This will allow us to determine if severity upon presentation makes a difference to when to resume anticoagulation/antiplatelet therapy (AAT) and, if so, what the difference in AAT would be based upon TBI severity.

## Conclusions

Based on our study results, we conclude that the best time to resume OAC in TBI is between seven and 14 days, particularly on Day 10. Study participants who resumed OAC in this range had the fewest number of adverse events measured at the six-month follow-up and the lowest mortality rate. However, due to the lack of statistical power in our study, we cannot make any definitive recommendations on the best time to resume OAC in TBI. We recommend a prospective study with one protocol on the resumption of OAC with a six-month follow-up to assess the outcomes.
